# AFND: Arabic fake news dataset for the detection and classification of articles credibility

**DOI:** 10.1016/j.dib.2022.108141

**Published:** 2022-04-08

**Authors:** Ashwaq Khalil, Moath Jarrah, Monther Aldwairi, Manar Jaradat

**Affiliations:** a Department of Computer Engineering, Jordan University of Science and Technology, PO Box 3030, Irbid 22110, Jordan; b College of Technological Innovation, Zayed University, Abu Dhabi, UAE; c Department of Computer Engineering, The Hashemite University, PO Box 330127, Zarqa 13133, Jordan

**Keywords:** Arabic news dataset, Arabic fake news, Article credibility, Weak labeling, Detection, Classification

## Abstract

The news credibility detection task has started to gain more attention recently due to the rapid increase of news on different social media platforms. This article provides a large, labeled, and diverse Arabic Fake News Dataset (AFND) that is collected from public Arabic news websites. This dataset enables the research community to use supervised and unsupervised machine learning algorithms to classify the credibility of Arabic news articles. AFND consists of 606912 public news articles that were scraped from 134 public news websites of 19 different Arab countries over a 6-month period using Python scripts. The Arabic fact-check platform, Misbar, is used manually to classify each public news source into credible, not credible, or undecided. Weak supervision is applied to label news articles with the same label as the public source. AFND is imbalanced in the number of articles in each class. Hence, it is useful for researchers who focus on finding solutions for imbalanced datasets. The dataset is available in JSON format and can be accessed from Mendeley Data repository.

## Specifications Table

**Table:** 

Subject	Computer Science
Specific subject area	Natural Language Processing, Text Classification, Arabic Language
Type of data	Text files
How the data were acquired	Scraping public news websites using Python scripts, Operating System (Ubuntu Linux)
Data format	JSON
Description of data collection	134 public Arabic news websites were selected randomly for article scrapping. The public websites cover 19 different Arab countries. The countries are Jordan, Lebanon, Egypt, Iraq, United Arab Emirates, Saudi Arabia, Kuwait, Qatar, Bahrain, Syria, Oman, Tunisia, Morocco, Algeria, Libya, Palestine, Sudan, Yemen, and Mauritania. Online news for Djibouti, Somalia, and Comoros were not included because of difficulties in finding their news sources and their labeling in the Misbar fact-check platforms. The language of the news articles is Modern Standard Arabic (MSA). Articles were scraped every day from the predetermined public news websites using Python scripts over six months. Feedparser [1] and newspaper [2] Python libraries were used to scrap the articles. All duplicated articles were removed. During that time, articles from the public news sources were also collected from the fact-checking platform Misbar [3]. The annotation criteria for articles in Misbar was used to classify each public news source into three classes which are: credible, not credible, or undecided. Each scrapped article was labeled according to its public source class.
Data source location	Institution: Jordan University of Science and Technology
	City/Town/Region: Irbid
	Country: Jordan
	Latitude and longitude (and GPS coordinates) for collected samples/data: 32.4913${}^{\circ }$ N, 35.9875${}^{\circ }$ E
Data accessibility	Repository name: Mendeley Data
	Data identfication number: 10.17632/67mhx6hhzd.1
	Direct URL to data: https://data.mendeley.com/datasets/67mhx6hhzd/1.
Related research article	A. Khalil, M. Jarrah, M. Aldwairi, Y. Jararweh, Detecting arabic fake news using machine learning, in: 2021 Second International Conference on Intelligent Data Science Technologies and Applications (IDSTA).(2021) 171-177. https://www.doi.org/10.1109/IDSTA53674.2021.9660811

## Value of the Data

• AFND is a large, diverse, single-labeled Arabic news dataset that is suitable for training deep learning models to detect Arabic fake news.
• Different natural language processing (NLP) techniques that focus on Arabic language processing can use the dataset to find errors and noise for correction.
• The dataset is convenient for both deep learning and conventional machine learning algorithms due to its size and diversity.
• Unsupervised machine learning algorithms can use the dataset to process and classify undecided news articles into credible or not credible.
• The dataset is imbalanced in the number of articles that belongs to each class. Also, there is a high variation in the number of tokens/words within articles. Therefore, different NLP techniques and machine learning algorithms can use the dataset to solve these problems.

## 1 Data Description

**Fig. 1:**
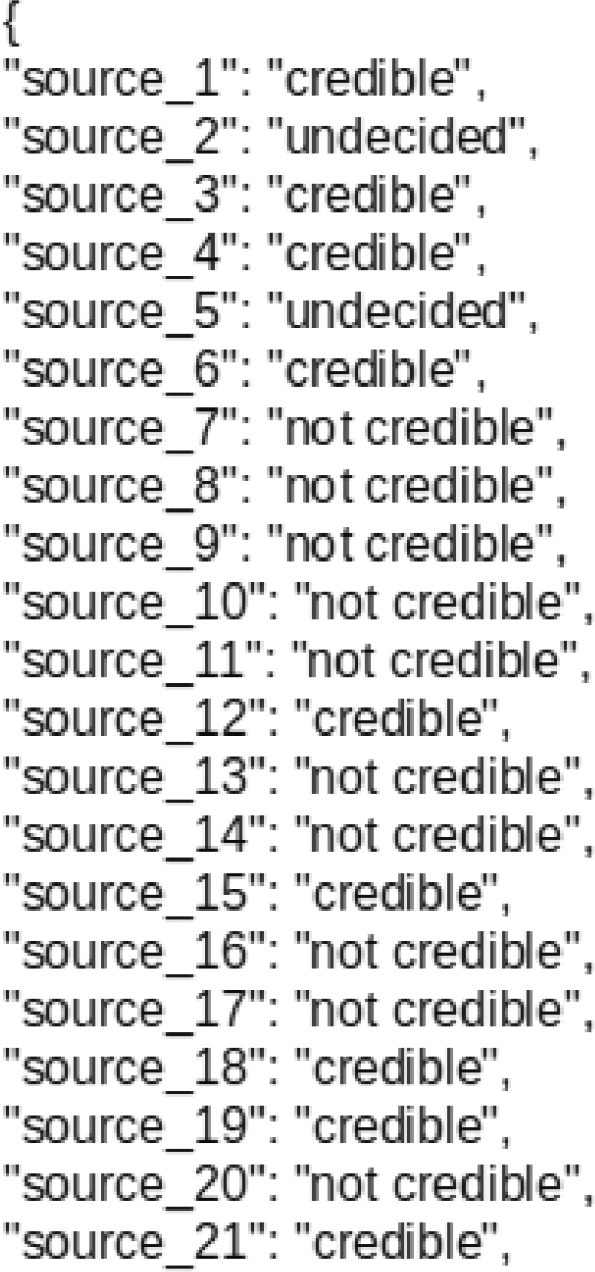
Example of the contents of the JSON file named “sources.json”.

Although the 134 news websites are free and public, we have replaced the identity (Uniform Resource Locator URL) of each website with “source_1”, “source_2”, “source_3”, etc. in the JSON objects in a file named “sources.json” under the main directory to anonymize the news source websites. Each object has an anonymous name of the news source and a label for the news source (credible, not credible, or undecided). Fig. 1 shows a portion of the contents of the file “sources.json”.

**Fig. 2:**
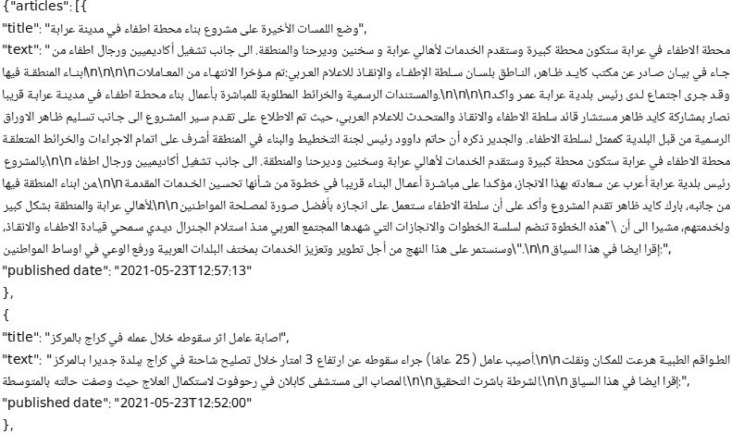
Example of a scraped article that is available in the public domain.

The scraped public Arabic articles are stored in 134 sub-directories within the directory named “Dataset”. Each sub-directory is named according to the anonymous names (e.g. “source_1”) of the 134 news sources. Each sub-directory has a file named “scraped_articles.json” which contains the information of the articles that were scraped from the public news source and stored as an array of JSON objects. Each object stores the title, text, and the publication date of the article. Fig. 2 shows an example of a JSON object for a scrapped article from an anonymous news source.

Table 1 presents the number of news sources and articles in each class, in addition to the average number of words in the title and body text of the scraped articles.

**Table 1:** Dataset statistics.

Statistical	Credible	Not Credible	Undecided
number of articles	207310	167233	232369
Number of Sources	52	51	31
Average number of words in body text	230	217	254
Average number of words in title	9	10	9

**Fig. 3:**
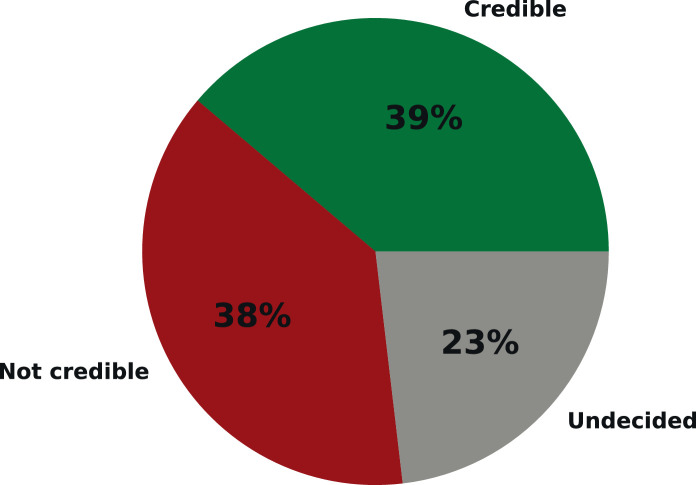
The percentage of public news sources in each class.

**Fig. 4:**
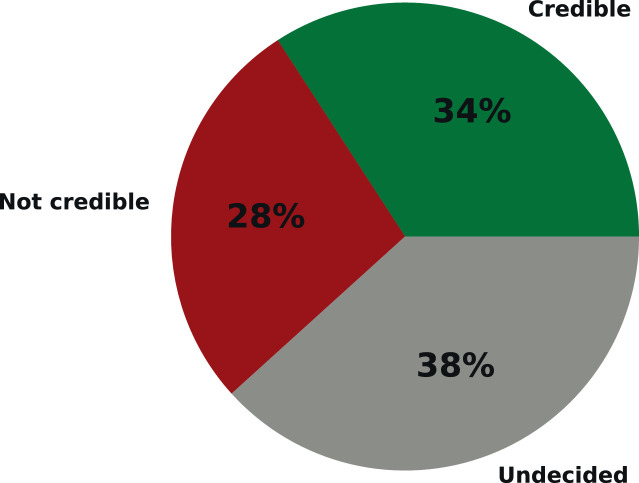
The percentage of public articles in each class.

**Fig. 5:**
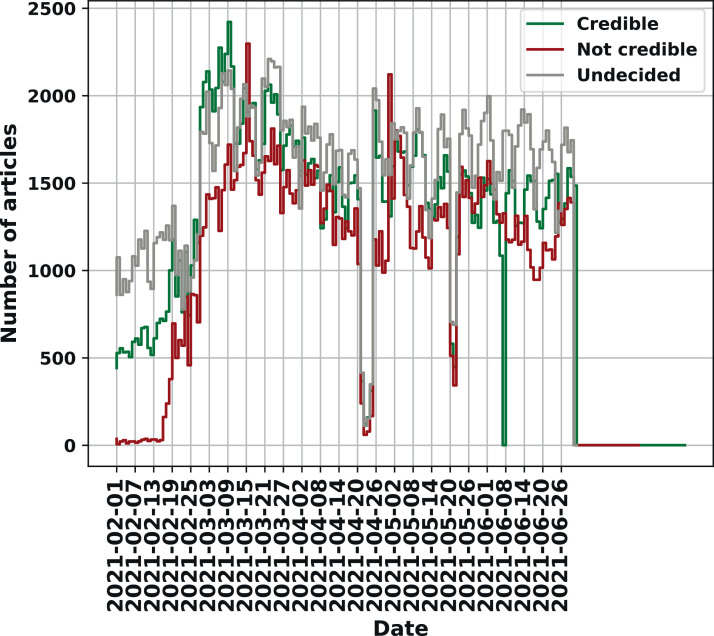
Timeline: the number of collected articles per day.

Figs. 3 and 4 show the percentage of public news sources and articles that belong to each class. Moreover, Fig. 5 presents the number of collected articles per day during the six month period of article scraping (from February 1, 2021 to July 30, 2021).

## 2 Experimental Design, Materials and Methods

Here, we present the issues that we encountered during the collection of the dataset and the methods that we used to handle them.

Many of the public news websites have separate link pages that are related to local news of some Arab countries. The local pages were selected rather than the main page for each source to avoid international news and to scrape more public news articles. Out of the 134 news sources, 82 public websites support the RSS feed of local news. Thereby, the public news articles for these sources were scraped from the RSS feed and the local page links.

The public news articles were scraped for six months starting from February 1, 2021 to July 30, 2021. During this period, we have continuously checked the collected public articles to ensure the soundness of the data scraping. Duplicated articles, unrelated articles of the public source (external links), articles in non-Arabic languages, articles supported by videos rather than text, and articles that are unrelated to events or news were all eliminated. Moreover, no pre-processing methods were performed on the articles text. It is worth mentioning that all these articles are available in the public domain.

Misbar, the public Arabic fact check platform, uses a rating system that labels published articles in public news websites. There are eight labels which are: True, Fake, Misleading, Selective, Suspicious, Myth, Commotion, and Satire. We used Misbar and searched manually for all Arabic news that were already labeled in the previous 9 months. Also, during the scraping phase, Misbar was continuously checked for new articles from any of the 134 public news websites. Some of these news articles were published by one or more of the chosen news websites. When all public news articles (based on Misbar) of a news website were classified as True, then the website was classified as credible. On the other hand, when all public news articles (based on Misbar) of a news website were not-True (belong to one of the remaining seven categories), then the website was classified as not credible. Finally, public news websites that have articles that belong to both categories (True and not-True) were classified as undecided. After that, all scraped articles from a credible websites were labeled as credible, scraped articles from not credible website were labeled as not credible, and scraped articles from an undecided website were labeled as undecided. This distant labeling method can result in errors and noise and our analysis has shown that. Hence, natural language processing techniques can be used by researchers to find errors and correct them.

We have investigated the AFND dataset and it is found that it is imbalanced in terms of the number of news websites and the number of articles in each class. The percentage of public websites within the undecided class is less than credible and not credible classes. However, the number of public articles that belong to the undecided class is the largest. This observation opens a research problem on using natural language processing techniques to mine the articles text to find correlations and semantic similarities that can automatically detect public Arabic fake news. The dataset was used in a research paper, titled “Detecting Arabic Fake News Using Machine Learning” [4], were different machine learning models were applied for Arabic fake news detection and classification.

## Ethics Statements

This work involved **no** human subjects, **no** animal experiments, and data was **not** collected from social media platforms.

• Terms of service (ToS): The public and free Arabic news websites are available for any one and allow the news articles to be scraped. We are not using the data for any fraudulent activities such as making profit (e.g.business), DDoS, data theft, or any other sort of bad intention.
• Copyright: The data does NOT belong to users (e.g. social media). The data are published on free and public news sites that can be consumed by any one who has access to the Internet. This is similar to search engines that use bots to index web pages.
• Privacy: Although the data is free and published in the public domain, we have replaced the identity (Uniform Resource Locator URL) of each website with “source 1”, “source 2”, “source 3”, etc. to anonymize the websites and the news articles.
• Scraping policies: There is no special scraping policy.

## CRediT authorship contribution statement

**Ashwaq Khalil:** Methodology, Software, Data curation, Formal analysis, Investigation, Validation, Writing – original draft, Visualization. **Moath Jarrah:** Conceptualization, Validation, Resources, Writing – review & editing, Supervision, Project administration, Funding acquisition. **Monther Aldwairi:** Conceptualization, Supervision, Funding acquisition. **Manar Jaradat:** Writing – review & editing.

## Declaration of Competing Interest

The authors declare that they have no known competing financial interests or personal relationships that could have appeared to influence the work reported in this paper.

## Data Availability

ArabicFakeNewsDataset (Original data) (Mendeley Data).
